# The Clinical Utility of Cell-Free DNA Measurement in Differentiated Thyroid Cancer: A Systematic Review

**DOI:** 10.3389/fonc.2018.00132

**Published:** 2018-04-30

**Authors:** Jonathan M. Fussey, Jennifer L. Bryant, Nikolaos Batis, Rachael J. Spruce, Andrew Hartley, James S. Good, Christopher J. McCabe, Kristien Boelaert, Neil Sharma, Hisham Mehanna

**Affiliations:** ^1^School of Cancer Sciences, Institute of Head and Neck Studies and Education, University of Birmingham, Birmingham, United Kingdom; ^2^Hall-Edwards Radiotherapy Research Group, Queen Elizabeth Hospital, Birmingham, United Kingdom; ^3^Department of Oncology, University Hospital Birmingham, Birmingham, United Kingdom; ^4^College of Medical and Dental Sciences, Institute of Metabolism and Systems Research, University of Birmingham, Birmingham, United Kingdom

**Keywords:** thyroid cancer, DNA, cell-free, cell-free DNA, circulating DNA, cell-free systems

## Abstract

**Background:**

Cell-free DNA (cfDNA) can be detected in the circulation of healthy individuals, but is found in higher concentrations in cancer patients. Furthermore, mutations in tumor cells can be identified in circulating DNA fragments. This has been the subject of significant interest in the field of cancer research, but little has been published in thyroid cancer.

**Objectives:**

To assess all available evidence on the use of circulating cfDNA in the diagnosis, management and surveillance of patients with differentiated thyroid cancer, and collate it into a systematic review to guide future research.

**Methods:**

A comprehensive literature search on the measurement of cfDNA in thyroid cancer was undertaken, and results from relevant studies collated into a systematic review.

**Results:**

Nine studies were identified, with varying methodologies and findings. Key techniques and findings are summarized.

**Conclusion:**

There is limited but promising evidence that somatic mutations in thyroid cancer can be detected in circulating cfDNA and are associated with more advanced disease. Further research is required to develop a clinically useful tool based on cfDNA to improve the management of thyroid cancers.

## Introduction

### Rationale

Differentiated thyroid cancer (DTC) accounts for around 1% of all human cancers (1), and rates of diagnosis have been increasing in recent years, in part due to improving imaging techniques (2). Although survival rates in DTC are very good, there remains a subset of patients in whom the cancer behaves more aggressively and requires more aggressive management and follow-up. Distinguishing between these patients and those with indolent DTC remains a challenge. Management of DTC includes thyroidectomy with or without postoperative radioiodine ablation and long-term thyroid stimulating hormone suppression (3). Surveillance of DTC patients following treatment relies in part on measurement of serum thyroglobulin levels. However, 25–30% of DTC patients have thyroglobulin antibodies (4), which limit the usefulness of this test.

Cell-free DNA (cfDNA) was first described in 1948 (5). Tumor DNA fragments are released into the circulation as a result of necrosis and apoptosis (6), or secretion by cancer cells. Modern polymerase chain reaction (PCR) and sequencing techniques allow identification of nucleic acids in peripheral blood samples, and thus represent an opportunity for the development of novel diagnostic and surveillance tests in cancer patients. Indeed, in other cancers, the measurement of cfDNA has been shown to be a feasible clinical tool (7, 8).

Many somatic mutations have been described in DTC, and some have been shown to predict more aggressive tumor behavior (9, 10). For example, a point mutation in the BRAF gene resulting in the substitution of valine for glutamate at codon 600 can be identified in 24.7–74.7% of papillary thyroid cancers depending on subtype (10). A large meta-analysis demonstrated statistically significant correlation between BRAF^V600E^ and tumor size, lymph node metastasis, multifocality, extrathyroid extension, and clinical stage (10).

### Objective

With the current challenges in risk stratification and surveillance of thyroid cancer patients, the possibility of detecting circulating tumor cfDNA represents a promising potential advance in the management of DTC. The objective of this review was to systematically review the current evidence on the subject of cfDNA measurement in DTC patients.

### Research Question

Can circulating cell-free tumor DNA be used to aid the diagnosis, risk stratification, and surveillance of patients with DTC?

## Materials and Methods

### Study Design and Search Strategy

A preliminary search of the Cochrane library was performed to identify pre-existing reviews on the subject, but none was found. A comprehensive search of the published literature was then performed using Medline. Search terms and Boolean operators used were “cell free systems” OR “cell free DNA” OR “cell free nucleic acids” OR “cell free RNA” OR “nucleic acids” OR “circulating DNA” AND “adenocarcinoma, papillary” OR “adenocarcinoma, follicular” OR “thyroid neoplasm.” Filters were used to identify articles concerning humans and published in the last 15 years. No language restrictions were applied. Titles and abstracts were screened for relevance according to a previously developed review protocol. Reference lists were manually searched for relevant articles not identified by the initial search. Finally, a generic Internet search engine was used to further maximize the search yield.

### Participants, Interventions, Comparators

All articles concerning the measurement of cfDNA in adult thyroid cancer patients were considered. Studies describing the use of cfDNA for diagnosis, prognosis, or surveillance were included. Articles that did not include patients with DTC were excluded, as were review articles and those with fewer than 10 subjects. No restrictions were placed on study design, outcome measures, or study population.

### Systematic Review Protocol and Data Extraction

Data were extracted from included studies and collated using a *pro forma* in Microsoft Excel (Redmond, WA, USA). Categories for data extraction included study design; objective for cfDNA measurement; demographic details of subjects; tumor histology and characteristics; method of cfDNA measurement; format of results reporting and quantitative and qualitative results.

### Data Analysis

Due to the heterogeneity of the data reported by different authors and the variation in outcome measures used, statistical meta-analysis was not possible. Therefore, following data extraction, results were grouped and reported according to outcome measure.

## Results

### Search Results and Study Selection

**Figure 1:**
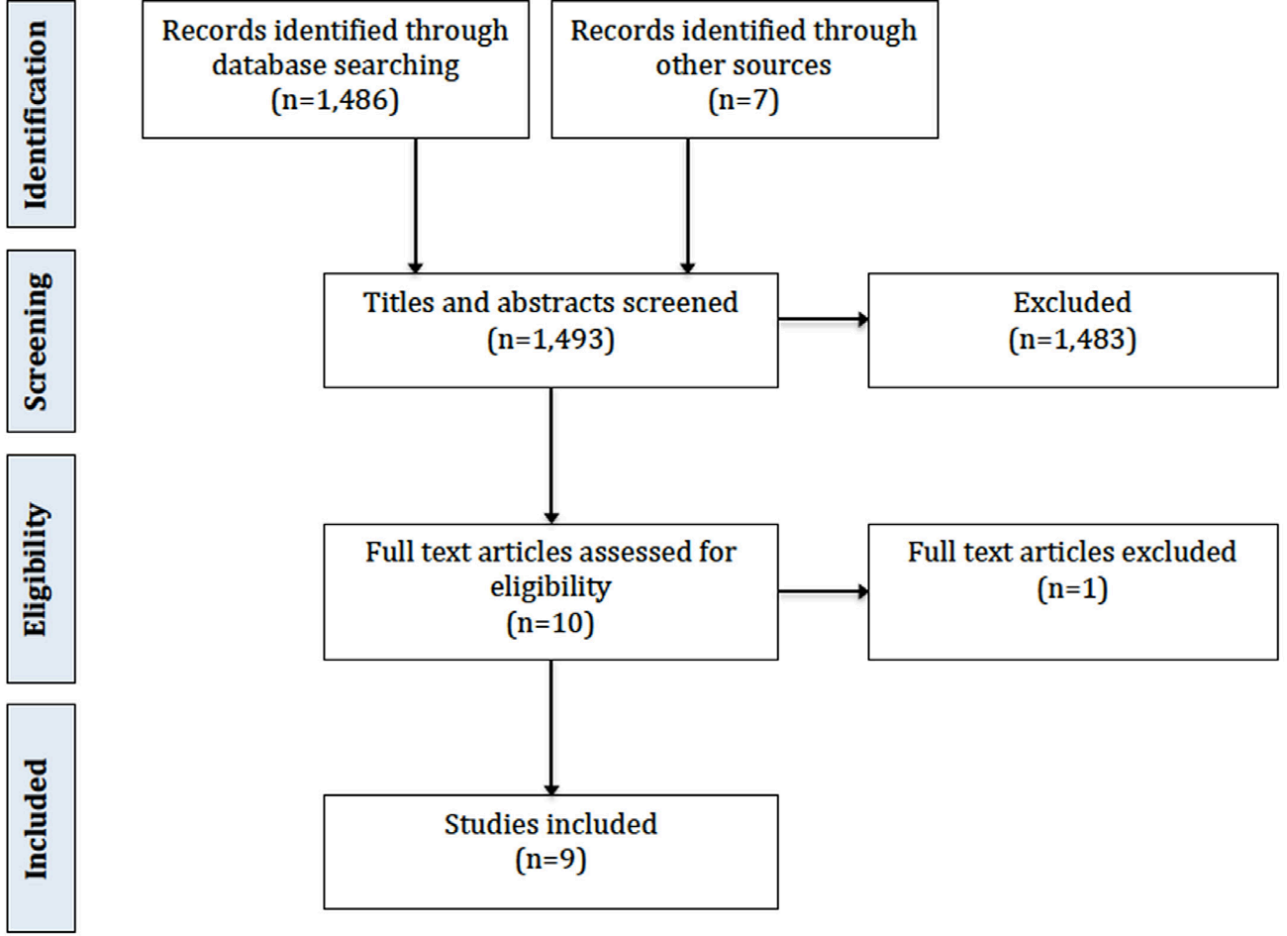
Preferred Reporting Items for Systematic Reviews and Meta-Analyses 2009 flow diagram.

The initial Medline search as described above produced 2,680 titles. This was reduced to 1,486 titles by applying filters for human studies published in the last 15 years. Of these, 1,483 could be excluded based on title and abstract alone due to irrelevance to the review objective. This left three articles for inclusion. In addition to this, a manual search of reference lists identified five further articles, and one further article and an unpublished thesis were identified using Internet search engines. This resulted in nine articles for inclusion. Figure 1 shows the Preferred Reporting Items for Systematic Reviews and Meta-Analyses flowchart.

### Characteristics of Included Studies

The nine included studies were published between 2006 and 2017. Eight of them were peer-reviewed publications (11–18) and one was a scientific thesis published on the Electronic Thesis and Dissertation Repository (19). Seven studies were prospective, and two were retrospective. There were a total of 994 patients in the combined studies, with an average of 110 (range 28–200) per study. Of these, 633 had confirmed DTC. Three studies included healthy subjects as controls, and seven included patients with benign thyroid disease. The studies were heterogeneous in their aims and objectives, with some setting out to use cfDNA measurement as a diagnostic tool, some investigating its utility as a prognostic marker, and others simply assessing feasibility of cfDNA measurement in thyroid cancer. There was also significant variation in the outcome measures reported, so meta-analysis was not appropriate. General study characteristics are represented in Table 2.

### cfDNA Measurement

All studies described the extraction of cfDNA in detail. Five studies used plasma samples, three used serum samples, and one used whole blood. Following DNA extraction, the majority of studies used real-time PCR to identify BRAF^V600E^. In some cases, this was quantitative TaqMan PCR with primers for both BRAF^V600E^ and BRAF^WT^ alleles, and in others, digestion of wild type alleles was used to reduce contamination by normal BRAF from surrounding tissues. In addition, two studies used quantitative methylation-specific PCR to identify methylation in target genes (14, 17). One study, by Salvianti et al., measured the percentage of fragments of differing lengths of the APP gene circulating in plasma, in the range of 67–180 base pairs. Using quantitative real-time PCR, they calculated the quantity of the longer and shorter fragments in plasma, then calculated the ratio between the absolute concentration of the longer and shorter fragments to give a cfDNA integrity index (18). They then measured this in thyroid cancer patients and healthy individuals to test the hypothesis that longer strand cfDNA can be used as a biomarker for tumor presence.

Three studies measured cfDNA in healthy individuals. Zane et al. found a statistically significantly lower level of cfDNA in healthy individuals compared with thyroid cancer patients (median levels 5.14 and 22.54 ng/ml, respectively, *p* < 0.0001) (17). Pupilli et al. found a statistically significantly elevated percentage of circulating BRAF^V600E^ mutated allele over wild type allele in patients with Thy3 (18.7%) and Thy4 and Thy5 (27.1%) cytology compared with healthy individuals (1.7%) (11). Salvianti et al. found both a lower level of cfDNA in healthy individuals, and a lower cfDNA integrity index when compared with patients with thyroid nodules of any cytology (18).

Seven of the nine studies included patients with benign thyroid disease. Two of them found no circulating BRAF^V600E^ in their benign patients (15, 19), and one was able to show a statistically significant difference in circulating BRAF^V600E^ levels between those with benign thyroid disease and thyroid cancer (11). Pupilli et al. demonstrated a circulating BRAF^V600E^ over BRAF^WT^ percentage of 9.9% in patients with benign thyroid disease and 27.3% in patients with cancer (11).

Overall six studies reported on the presence of the BRAF^V600E^ cfDNA mutation in DTC patients, with a combined total of 438 affected individuals (11, 13, 15–17, 19). Circulating BRAF^V600E^ was measured by some authors pre-treatment and by others post-treatment, and was identified in 10% of these patients overall. Of the 302 patients in whom tumor BRAF^V600E^ was measured, 36.8% were positive. The proportion of patients with BRAF^V600E^ mutation in the tumor who also had circulating BRAF^V600E^ was 16.5%.

### cfDNA as a Diagnostic Tool

Pupilli et al. prospectively measured the percentage of plasma BRAF^WT^ and BRAF^V600E^ cfDNA in 38 patients with PTC, 16 with benign disease, and 49 healthy individuals (11). They compared levels of circulating BRAF^V600E^ with cytology results and with final histology. They found that patients with Thy2 cytology had a significantly lower percentage of BRAF^V600E^ cfDNA (8.9%) than those with Thy4 and Thy5 cytology (27.1%). They also demonstrated a statistically significantly elevated circulating BRAF^V600E^ level in patients with PTC (23.7%) compared with those with benign thyroid nodules (9.9%).

Hu et al. measured methylation of cfDNA, specifically focusing on five genes: CALCA, CDH1, TIMP3, DAPK, and RARβ2 (14). They retrospectively compared methylation levels in peripheral blood samples taken at the time of nodule assessment between thyroid cancer patients and those with benign thyroid nodules. When all five genes were considered, they achieved a diagnostic sensitivity of 68%, and a specificity of 95%, with a preoperative diagnostic accuracy of 77%. They also looked at a subset of patients with indeterminate cytology, and found that none of the four patients who were eventually found to have benign disease had positive cfDNA methylation levels, whereas 8 of the 11 patients (73%) who were later found to have cancer had elevated cfDNA methylation levels. Salvianti et al. were able to demonstrate a statistically significant difference in cfDNA integrity index between those with Thy2 nodules and those with Thy 3 and Thy4/5 nodules (0.67, 0.83, 1.02, respectively, *p* < 0.001) (18).

### cfDNA as a Marker of Advanced Disease

Several of the studies investigated the potential usefulness of cfDNA measurement in the identification of patients with advanced locoregional or metastatic disease. Kim et al. were only able to identify BRAF^V600E^ cfDNA in 3 of 72 PTC patients, but all three of them had lateral neck node metastasis and lung metastasis (15). They therefore postulated that circulating BRAF^V600E^ might have a role in predicting lung metastasis. Patel investigated the role of circulating BRAF^V600E^ in predicting stage of disease in a sample of six patients, but found no statistically significant association between detectable BRAF^V600E^ cfDNA and primary tumor T classification, nodal metastasis, or extrathyroidal extension (19). Similar methods of DNA extraction were used by both authors, which suggest a statistical cause for divergent results.

### cfDNA as a Marker for Monitoring of Disease Recurrence

Four studies examined the feasibility of the use of cfDNA for monitoring of disease recurrence in thyroid cancer patients. Hu et al. retrospectively measured cfDNA methylation levels of a five-gene panel at post-treatment visits in patients being followed up after treatment for DTC. They correctly identified 70% of those with proven recurrence by gold standard techniques including radioiodine body scan, tissue biopsy and stimulated thyroglobulin, and 79% of those with no evidence of recurrence (14). Pupilli et al. found a statistically significant drop in BRAF^V600E^ cfDNA levels following treatment, with 71% of patients who were preoperatively positive for circulating BRAF^V600E^ becoming negative following treatment (12). They suggest that it could be a useful tool in surveillance post-treatment, but warned that further research is required. Cradic et al. found that patients with detectable circulating BRAF^V600E^ following treatment had a relative risk of 2.55 (*p* < 0.04) of having recurrent disease at the time of blood draw compared with those with only BRAF^WT^ detected in peripheral blood (13). Salvianti et al. measured plasma cfDNA integrity index in 17 patients 3–6 months following surgery with or without radioiodine ablation, and found a lower index post treatment (0.57) than before treatment (0.87, *p* = 0.35) (18).

### Risk of Bias and Quality Assessment

All studies were assessed for quality and risk of bias according to the modified Quality Assessment of Diagnostic Accuracy Studies checklist (20) (Table 1). All papers were deemed to have a low overall risk of bias, however, only one explicitly described consecutive patient recruitment, and only three described a pre-specified threshold for a positive result.

**Table 1:** Quality Assessment of Diagnostic Accuracy Studies-2 checklist [adapted from Whiting et al. (20)].

**Domain 1: Patient Selection**
Was a consecutive or random sample of patients enrolled? (Yes/No/Unclear)
Was a case-control design avoided? (Yes/No/Unclear)
Did the study avoid inappropriate exclusions? (Yes/No/Unclear)
Could the selection of patients have introduced bias? Risk: Low/High/Unclear

**Domain 2: Index Test(s) (complete for each index test used)**
Were the index test results interpreted without knowledge of the reference standard? (Yes/No/Unclear)
If a threshold was used, was it pre-specified? (Yes/No/Unclear)
Could the conduct or interpretation of the index test have introduced bias? Risk: Low/High/Unclear

**Domain 3: Reference Standard**
Is the reference standard likely to correctly classify the target condition? (Yes/No/Unclear)
Were the reference standard results interpreted without knowledge without knowledge of the results of the index test? (Yes/No/Unclear)
Could the reference standard, its conduct, or its interpretation have introduced bias? Risk: Low/High/Unclear

**Domain 4: Flow and Timing**
Was there an appropriate interval between index test(s) and reference standard? (Yes/No/Unclear)
Did all patients receive a reference standard? (Yes/No/Unclear)
Did all patients receive the same reference standard? (Yes/No/Unclear)
Were all patients included in the analysis? (Yes/No/Unclear)
Could the patient flow have introduced bias? Risk: Low/High/Unclear

**Table 2:** General study characteristics.

Reference	Year of publication	Study design	No. of patients	cfDNA measurement	Key findings
Pupilli et al. (11)	2013	Prospective	103	Plasma cfDNA BRAF^V600E^	Percentage of BRAF^V600E^ significantly higher in those with PTC. Significant drop after treatment

Chuang et al. (12)	2010	Prospective	28	Serum cfDNA BRAF^V600E^	Of those with tumor BRAF^V600E^, 60% also had detectable cfDNA BRAF^V600E^

Cradic et al. (13)	2009	Prospective	193	Circulating BRAF^V600E^	BRAF^V600E^ detected in blood of 11.6% of DTC patients and correlated with active disease

Hu et al. (14)	2006	Retrospective	92	Serum cfDNA methylation various genes	cfDNA methylation of β-actin, CALCA, CDH1, TIMP3, DAPK, RARβ2 in 95% DTC with 96% PPV

Kim et al. (15)	2015	Retrospective	77	Plasma cfDNA BRAF^V600E^	cfDNA BRAF^V600E^ mutation only identified in 4.2% of PTC patients, but all of these had lung metastasis

Kwak et al. (16)	2013	Prospective	94	Serum cfDNA BRAF^V600E^	Unable to identify BRAF^V600E^ mutation in the serum of any individual with PTC

Zane et al. (17)	2013	Prospective	200	Plasma cfDNA BRAF^V600E^ and methylation of SLC5A8 and SLC26A4	Higher levels of cfDNA in DTC patients, but unable to isolate BRAF^V600E^ in circulation in any patients

Salvianti et al. (18)	2017	Prospective	146	Plasma cfDNA and APP gene integrity index	Correlation between cfDNA integrity index and cytological evidence of thyroid cancer. Reduction in integrity index following treatment

Patel (19)	2015	Prospective	61	Plasma cfDNA BRAF^V600E^	cfDNA BRAF^V600E^ in 23% of those with PTC and none of those with benign nodules. Levels fell post-treatment

*PTC, papillary thyroid carcinoma; DTC, differentiated thyroid carcinoma; PPV, positive predictive value; cfDNA, cell-free DNA*.

## Discussion

### Summary of Main Findings

This review highlights the potential usefulness of cfDNA in thyroid cancer patients, and therefore justifies further research given the relative lack of evidence compared with other cancers. There has been significant interest in cfDNA in other cancers over the last 20 years, and it has been studied in breast, colorectal, pancreatic, and ovarian cancers, as well as brain tumors and melanoma (8, 21–25).

The vast majority of the existing evidence relates to the measurement of BRAF^V600E^ cfDNA. This is understandable as BRAF^V600E^ is the commonest and most investigated somatic mutation in DTC (26), and would therefore be a useful marker if it could be reliably detected in the circulation.

The BRAF^V600E^ mutation has been investigated in a number of human cancers, and while it is most commonly expressed in melanoma and thyroid cancer, circulating BRAF^V600E^ has also been found to be detectable in lung cancers (27). There is some evidence that the BRAF^V600E^ mutation in thyroid cancer is associated with more advanced disease, with increased likelihood of nodal and distant metastasis (28, 29).

While the majority of research in thyroid cancer cfDNA has focused on PCR identification of specific point mutations in circulating DNA, two studies have measured methylation of specific genes in cfDNA. Methylation is argued to be a more reliable marker due to its relative stability in cfDNA compared with point mutations, and the fact that there are a wide variety of mutations implicated in each cancer type, each with a relatively low frequency (30).

Of the studies that reported overall detection rates of BRAF^V600E^ in circulating cfDNA, the average detection rate in patients with DTC was 10%. This increased to 19.3% when patients with non-BRAF^V600E^ tumors were excluded. These relatively low levels may be due to the fact that tumors of all stages were included, and shedding of tumor DNA into the circulation is likely to occur at low levels in early stage disease and therefore may not be detectable with current techniques. The most promising evidence for the potential use of cfDNA as a diagnostic tool in thyroid cancer comes from Pupilli et al., who demonstrated a significantly higher proportion of BRAF^V600E^ cfDNA in patients with PTC than those with benign nodules, and a higher proportion in those with suspicious cytology than those with benign cytology (11). They suggest that the use of assay reagents with inadequate sensitivity could explain the variation in detection rates amongst other researchers. Their findings point to the feasibility of the developing a tool to help distinguish between benign and malignant nodules in patients with indeterminate cytology, or those with large nodules in whom a representative cytology sample is difficult.

It has been shown in other cancers that cfDNA levels correlate with stage of disease, and the rate of release into the circulation of cfDNA corresponds to primary tumor size (31, 32). In fact, one study has suggested that BRAF^V600E^ cfDNA levels are associated with nodal and distant metastasis in PTC (15), and a meta-analysis has demonstrated advanced clinical stage associated with BRAF^V600E^ mutation (10). This raises the potential of a peripheral detection of BRAF^V600E^ as a non-invasive marker of aggressive disease. cfDNA also represents a promising target for the development of new methods of detecting disease recurrence following treatment. Currently, surveillance depends on ultrasonography of the neck, and serial thyroglobulin measurement, which is hindered by the presence of antibodies in 25–30% of patients (4). Researchers studying other cancers have demonstrated that a drop in the level of total cfDNA during treatment can predict response to therapy (21), and this was also the case in the study that measured pre- and post-treatment levels in thyroid cancer (11).

### Limitations

Although cfDNA research is a rapidly evolving field, there is still a limited amount of evidence available relating to its use in thyroid cancer. The small number of published studies is a significant limitation on the ability of this systematic review to answer the research question.

There is also difficulty in comparing findings of studies on cfDNA in general due to various methods of DNA isolation and detection, and results reporting, which is also the case when considering the evidence in thyroid cancer. The use of serum and plasma measurements are described, and some authors report percentage of patients with detectable levels, some report quantitative levels and others report ratio of mutant to wild type alleles.

The majority of research to date has focused on the detection of one specific mutation in cfDNA—BRAF^V600E^. While BRAF is the most commonly mutated gene in sporadic PTC, with a rate of 18–87% (33, 34), The Cancer Genome Atlas project has shown the genomic landscape of PTC to be much more complex, with a number of significantly mutated genes (35). While the use of cfDNA for post-treatment surveillance has the advantage of the availability of somatic mutations in the resected tumor, if it were to become a sensitive diagnostic tool, expanded panels of genetic mutations would need to be detected to avoid false negatives in BRAF negative caners. Furthermore, there has been significant variation in the reported rates of detection of cfDNA in thyroid cancer patients. This could be due to differences in tumor expression of BRAF^V600E^ in different populations, differences in ability to detect cfDNA with different techniques, or non-representative study populations skewed by over-representation of early or advanced tumors. Further improvements and standardization in methodology are also needed. The use of a cfDNA integrity index as described by Salvianti et al. (18), which qualitatively evaluates cfDNA as well as measuring absolute concentrations, may prove to be a valuable addition to other biomarkers using cfDNA in thyroid cancer.

### Conclusion

Cell-free DNA measurement is an area of particular interest in the field of thyroid cancer management due to the current difficulties in diagnosis, identification of high-risk patients, and post-treatment surveillance. Early findings, although heterogeneous, provide promising insights and certainly warrant further investigation. A deeper understanding of how and when tumor DNA is shed from primary tumors and metastases into the circulation, and the development of more sensitive cfDNA assays are likely to improve progress toward the ultimate goal of a clinically useful test.

## Author Contributions

HM and NS conceived the study. JF devised and performed the search strategy. All authors were involved in preparation and approval of the final manuscript.

## Conflict of Interest Statement

The authors declare that the research was conducted in the absence of any commercial or financial relationships that could be construed as a potential conflict of interest.
